# Open-source Electronic Mass–Spring–Damper emulator with nonlinear dynamics for education and control research

**DOI:** 10.1016/j.ohx.2026.e00785

**Published:** 2026-05-08

**Authors:** Ernesto V. Gonzalez-Solis, David I. Rosas-Almeida

**Affiliations:** Facultad de Ingeniería, Universidad Autónoma de Baja California, Mexicali, Mexico

**Keywords:** Analog computing, Continuous-time systems, Nonlinear friction, Dead-zone nonlinearity, Hardware-in-the-loop, Education laboratory platforms

## Abstract

This work presents an open-source Electronic Mass–Spring–Damper (EMSD) emulator designed to reproduce the dynamic behavior of a second-order mechanical system in continuous-time and real-time operation. The proposed hardware emulates both linear dynamics and nonlinear effects commonly encountered in practical systems, such as dry friction and asymmetric dead-zone nonlinearities.

The emulator is realized using analog processing blocks that solve the associated nonlinear differential equation, avoiding numerical discretization and digital computation. Equivalent physical parameters such as mass, damping, stiffness, and nonlinear characteristics are adjusted through user-accessible front panel controls, enabling intuitive interaction and rapid reconfiguration.

The EMSD emulator provides analog input and output signals, allowing straightforward integration with external controllers, data acquisition systems, and hardware-in-the-loop (HIL) applications. Complete design files, build instructions, and documentation are provided to support reproducibility. The proposed platform offers a compact, low-cost, and transparent solution for hands-on education and experimental research in system dynamics and control, bridging the gap between numerical simulations and physical mechanical prototypes.

## Graphical abstract

**Figure:**
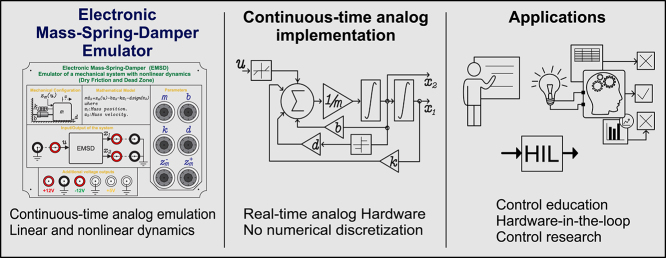

## Specifications table

**Table:** 

**Specifications table**	

Hardware name	Open-source Electronic Mass–Spring–Damper emulator with nonlinear dynamics for education and control research.

Subject area	• Engineering-Control Systems. • Electronics. • Educational hardware. • Automation and instrumentation.

Hardware type	• Analog electronic emulator for dynamics systems (mass–spring–damper). • Educational and research platform for the design and validation of control systems • Low-cost laboratory equipment.

Closest commercial analog	No commercial analog is available.

Open source license	The hardware presented in this work is released under the CERN Open Hardware License version 2 – Permissive (CERN-OHL-P). The associated software is distributed under the MIT License. All documentation, including design files and written materials, is made available under the Creative Commons Attribution 4.0 International License (CC BY 4.0).

Cost of hardware	*278.5 USD.*

Source file repository	https://doi.org/10.17605/OSF.IO/GD3Z8

	

## 1 Hardware in context

In science and engineering, access to experimental platforms is essential for connecting mathematical models to the behavior of dynamic systems. Control engineering, in particular, involves the development of mathematical models, the analysis of system dynamics, the design of control laws, and finally, the validation of closed-loop performance through experimentation [1], [2]. However, in many undergraduate and graduate programs — particularly in developing countries — this last step is often omitted due to the limited availability and high cost of laboratory equipment [3], [4]. Consequently, teaching tends to be highly dependent on numerical simulations, depriving students of practical challenges and the knowledge that arises from interacting with real systems [5].

In the field of control test benches and hardware-in-the-loop (HIL) systems, works such as [6] introduce a control strategy that emulates transfer functions to test mechanical actuators in a software-based linear mass–spring–damper system, allowing the dynamics of a physical system to be emulated in a way that reduces undesirable system effects and improves both fidelity and stability in HIL testing. Additionally, commercially available platforms based on mechanical assemblies often exhibit uncontrolled nonlinearities such as friction, backlash, dead zones, and actuator limitations—effects that hinder the validation of control algorithms, especially in the early stages of learning. Although these nonlinearities are relevant for advanced research, they may obscure the connection between theory and experimentation for beginners in the field. Virtual laboratories and numerical simulation tools can complement instruction [7], [8], [9], but cannot fully replace the need for physical experimentation [10].

Currently, the lack of accessible laboratory equipment for experimentation remains a persistent obstacle in science education. Commercially available materials and equipment for practical experimentation are often expensive and beyond the reach of many educational institutions [11], as they require considerable installation space, skilled labor for operation and maintenance, and some operating conditions that are difficult to reproduce in teaching environments. As a solution to this issue, several authors propose building low-cost didactic prototypes that allow the exploration of system dynamics using affordable components [12], [13], [14].

In addition to the previously mentioned approaches, different technological alternatives have been used for the study and implementation of dynamic systems. Traditional laboratory setups based on physical mass–spring–damper systems provide intuitive insight into system behavior; however, they suffer from drawbacks such as mechanical wear, parameter uncertainty, and limited flexibility for rapid reconfiguration. An example is described in US3483951 A [15], which focuses on vibration control in mechanical systems, considering the difficulties that these physical systems involve.

On the other hand, digital real-time simulators and numerical tools (e.g., MATLAB/Simulink based platforms or commercial systems such as dSPACE real-time simulators, including MicroLabBox, MicroAutoBox, and DS1104 board; and National Instruments platforms such as PXI, CompactRIO, and myRIO) offer high modeling flexibility and ease of implementation. However, these approaches rely on time discretization and numerical integration, which may introduce delays, quantization effects, and reduce transparency in the physical interpretation of system dynamics. Furthermore, commercial real-time platforms typically involve high acquisition and maintenance costs, which can limit their accessibility, particularly in educational environments with constrained budgets. Additionally, some methods are restricted to purely numerical simulation frameworks, as in CN101414314 [16], which focus on algorithmic solutions rather than physical implementation.

Analog computing platforms represent another alternative, as they enable continuous-time operation and direct implementation of differential equations. The knowledge in this field has a long and well-established history in the analysis and simulation of dynamical systems. Early analog computers were widely used throughout the mid-20th century for solving differential equations in real-time by exploiting the physical behavior of electronic circuits, particularly through the use of operational amplifiers, integrators, and function generators. As discussed in classical and modern Refs. [17], [18], [19], these systems provide an intuitive and direct mapping between mathematical models and physical signals, making them especially valuable for control engineering and educational purposes.

More recently, there has been renewed interest in analog and hybrid analog-digital computing platforms for modeling nonlinear systems, as highlighted in works such as [20]. These approaches demonstrate that analog computation remains a relevant paradigm, particularly for real-time simulation and HIL applications. Examples of programmable analog computers are described in US4074113A [21], as well as hybrid analog-digital systems like ES2316231B1 [22] or educational emulation platforms such as ES2167209B1 [23].

Compared to digital real-time simulators, the emulator operates entirely in continuous time, avoiding discretization effects and enabling direct interaction with analog signals and external control hardware without computational delays. Furthermore, unlike general purpose analog or hybrid systems, the proposed platform is specifically designed to emulate mass–spring–damper dynamics, including the structural integration of nonlinear effects such as dry friction and dead-zone characteristics.

The proposed hardware enables the emulation of a second-order system with configurable nonlinearities, providing a reproducible and fully electronic alternative with access to all input and output variables. This makes it a stable and predictable platform suitable for experiments in differential equations, transfer function identification, frequency response analysis, PID controller tuning, state-feedback control, and state observer design.

The emulator is implemented as a modular platform in which parameters such as mass, stiffness, and damping can be adjusted through independent controls. Its block-based circuit architecture simplifies replication and maintenance while facilitating integration with external hardware such as measurement instruments, function generators, analog controllers, or computer-based data acquisition systems. Consequently, the system offers a flexible, extensible, and easily configurable platform for a wide range of educational and research applications.

## 2 Hardware description

### 2.1 System overview

The proposed device is an open-source Electronic Mass–Spring–Damper (EMSD) emulator designed to emulate the behavior of a mass–spring–damper mechanical system in continuous-time and real-time operation, including nonlinear terms commonly encountered in real systems. The emulator is based on a fully analog architecture, enabling continuous-time operation without discretizations or computational delays. The emulator dynamics are implemented using continuous-time analog processing blocks that reproduce the behavior of the emulated mechanical system. The equivalent physical parameters are configured through user-adjustable front-panel controls.

**Fig. 1:**
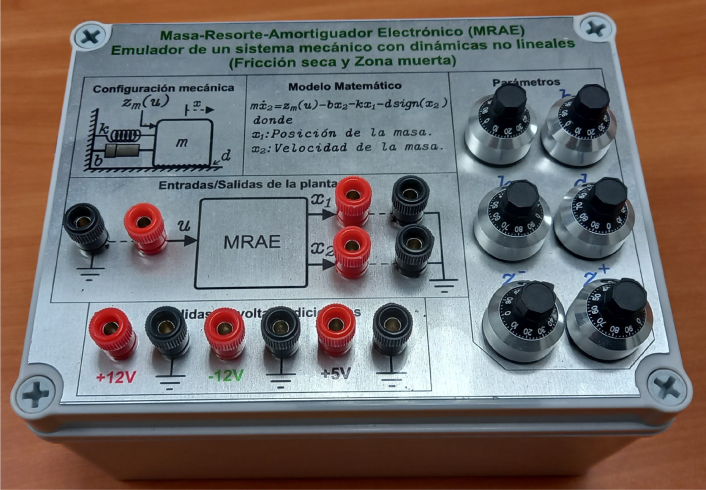
Assembled EMSD emulator showing the front panel, user adjustable parameters and signal terminals. Note: Labels shown in the prototype correspond to the original hardware implementation and are presented in Spanish.

Fig. 1 shows the assembled EMSD emulator corresponding to a representative hardware realization. The device integrates analog processing circuitry, a user interface, and signal terminals within a compact enclosure suitable for laboratory use. The front panel provides access to the adjustable parameters and signal connections required for experimental operation.

The EMSD emulator accepts an external analog excitation signal and generates analog output signals that represent the system response. The emulator provides an electronic platform for studying both linear and nonlinear dynamics without relying on numerical simulations or physical mechanical components. The device is intended for educational and experimental use in system dynamics and control, enabling intuitive interaction with the emulated physical parameters.

**Fig. 2:**
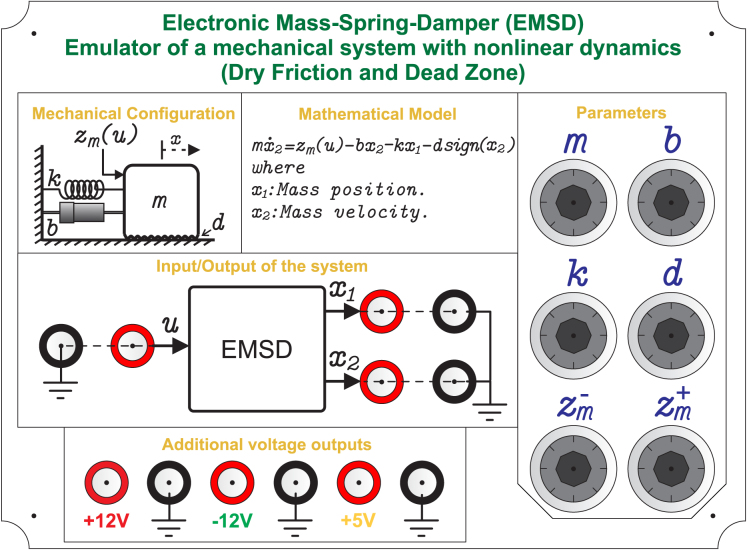
Front panel of the EMSD emulator, providing a functional overview of the system, including input/output terminals and adjustable parameters.

Fig. 2 shows the cover of the front view of the EMSD emulator, which contains the necessary information for the user to operate the hardware, such as input/output terminals and parameter adjustment elements.

### 2.2 Emulated mechanical system

The emulator is based on a classical mass–spring–damper mechanical configuration, augmented with nonlinear effects commonly encountered in practical systems, such as dry friction and a dead-zone with asymmetric limits.

**Fig. 3:**
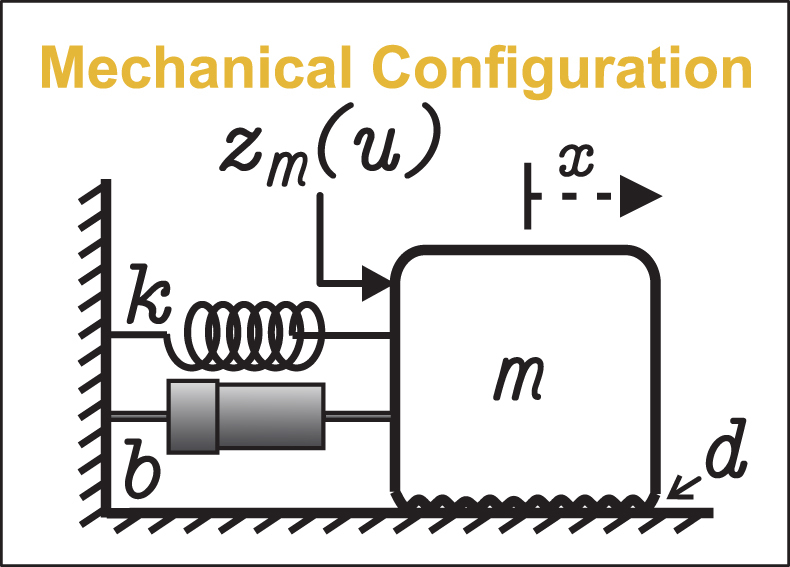
Conceptual representation of the mechanical mass–spring–damper system emulated by the proposed hardware.

Fig. 3 illustrates the mechanical configuration emulated by the proposed hardware. This conceptual representation provides the physical interpretation of the variables and parameters reproduced electronically.

### 2.3 Input and output signals

The EMSD emulator is designed to interact directly with external signal sources, measurement instruments, and control systems. The analog input signal represents an external excitation applied to the emulated mechanical system. The emulator produces analog output signals corresponding to the response of the system.

**Fig. 4:**
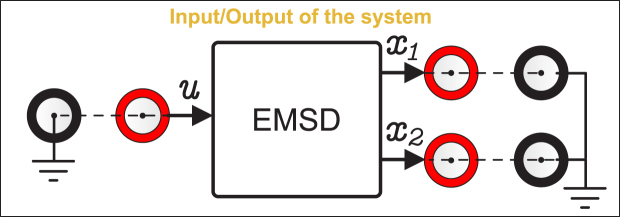
Block diagram showing the relationship between the input and the output signals of the EMSD emulator.

Fig. 4 shows a block diagram that describes the relationship between the input excitation signal and the available output signals of the EMSD emulator. This configuration enables straightforward integration of the device into experimental setups, including real-time control and HIL applications.

### 2.4 Mathematical model and analog implementation

Although the mathematical model of the system is illustrated in the front panel (see Fig. 2), a more detailed description is provided in this section to clarify its implementation using analog hardware.

The dynamics of the emulated system, shown in Fig. 3, are based on a second-order mass–spring–damper model with nonlinear effects, described by:

(1) $$m\overset{¨}{x}=z_{m}\left(u\right)-b\overset{˙}{x}-kx-d\;\mathrm{sign}\left(\overset{˙}{x}\right),$$

whose representation in state variables is

(2) $${\overset{˙}{x}}_{1}=x_{2},$$

$${\overset{˙}{x}}_{2}=\frac{1}{m}\left(z_{m}\left(u\right)-bx_{2}-kx_{1}-d\;\mathrm{sign}\left(x_{2}\right)\right),$$

where $x_{1}\left(t\right)$ represents the mass position, $x_{2}\left(t\right)$ its velocity, m is the mass, b is the viscous damping coefficient, k is the spring stiffness, and d is the dry friction coefficient. The term $z_{m}\left(u\right)$ represents a nonlinear input function that incorporates the dead-zone nonlinearity, considering asymmetric thresholds.

This formulation facilitates a direct mapping between the mathematical model, the functional block diagram, and the analog circuit implementation described in the following figures.

**Fig. 5:**
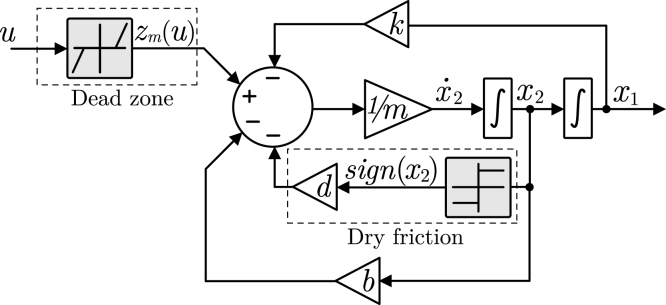
High-level functional block diagram of the EMSD emulator, showing the internal signal flow and main functional modules.

To provide a clearer understanding of the system architecture, Fig. 5 presents a high-level functional block diagram, where the dynamic equation (Eq. (2)) is decomposed into basic operations that can be implemented using analog circuits. As shown in this diagram, the input signal is first processed through the dead-zone nonlinearity and then combined with feedback terms corresponding to stiffness, damping, and dry friction in a summation stage. The resulting signal is scaled by the inverse of the mass and passed through two cascaded integrators to obtain the velocity and position signals. The nonlinear modules corresponding to the dead-zone and dry friction are explicitly highlighted, emphasizing their role in the dynamic system.

**Fig. 6:**
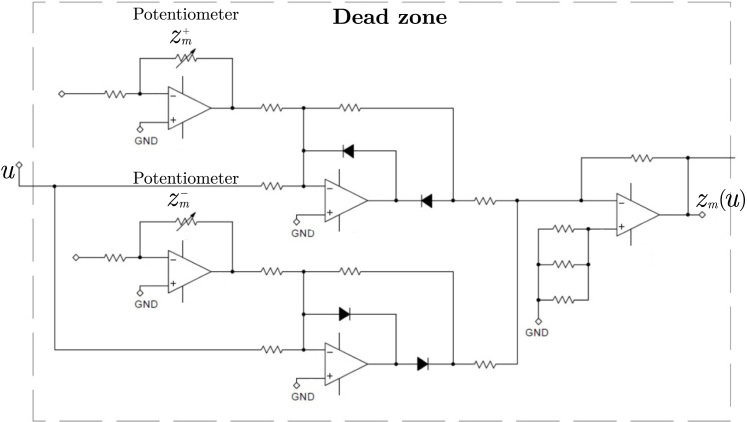
Electronic implementation of the dead-zone block.

**Fig. 7:**
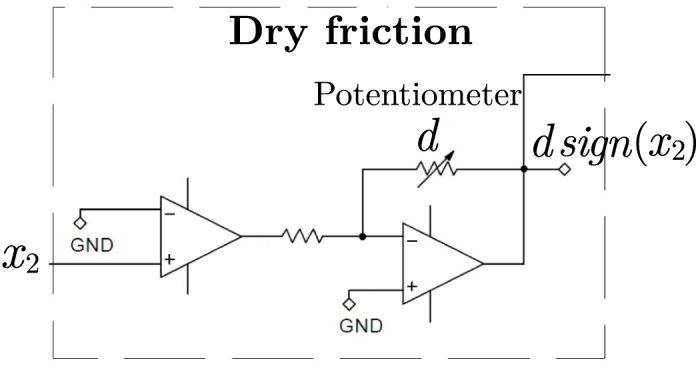
Electronic implementation of the dry friction block.

The relationship between this high-level representation and its circuit-level implementation is illustrated in Fig. 6, Fig. 7, where each nonlinear block is mapped to its corresponding analog circuit realization.

The term $z_{m}\left(u\right)$ is implemented using operational amplifiers and diodes. The input signal u is applied to a nonlinear switching stage that suppresses the output when the input remains within a predefined interval around zero, through the thresholds $z_{m}^{+}$ and $z_{m}^{-}$ adjusted via potentiometers, producing an output consistent with the dead-zone function.

Similarly, the dry friction module is implemented using operational amplifiers. The input signal $x_{2}$ is applied to a zero-crossing comparator, detecting the sign of the velocity signal. Its output is scaled by a gain stage controlled by a potentiometer that adjusts the friction coefficient d, producing a signal consistent with the term $d\;\mathrm{sign}\left(x_{2}\right)$.

This implementation establishes a direct correspondence between the mathematical model, its block diagram representation, and the analog hardware. Each term of the differential equation (Eq. (2)) is physically realized by a dedicated circuit module: linear terms such as inertia, damping, and stiffness are implemented using gain stages and integrators, while nonlinear effects such as dry friction and dead-zone are realized through dedicated nonlinear circuits. This one-to-one mapping enhances the intuitive understanding of system dynamics by directly linking mathematical models to measurable electrical signals.

### 2.5 Adjustable parameters and user interface

The emulator includes a front panel interface that allows the user to continuously adjust the equivalent physical parameters of the emulated system. These adjustable parameters enable the exploration of different dynamic behaviors and facilitate hands-on experimentation.

The user interface is designed to be intuitive, making the EMSD emulator suitable for educational environments as well as research applications.

**Fig. 8:**
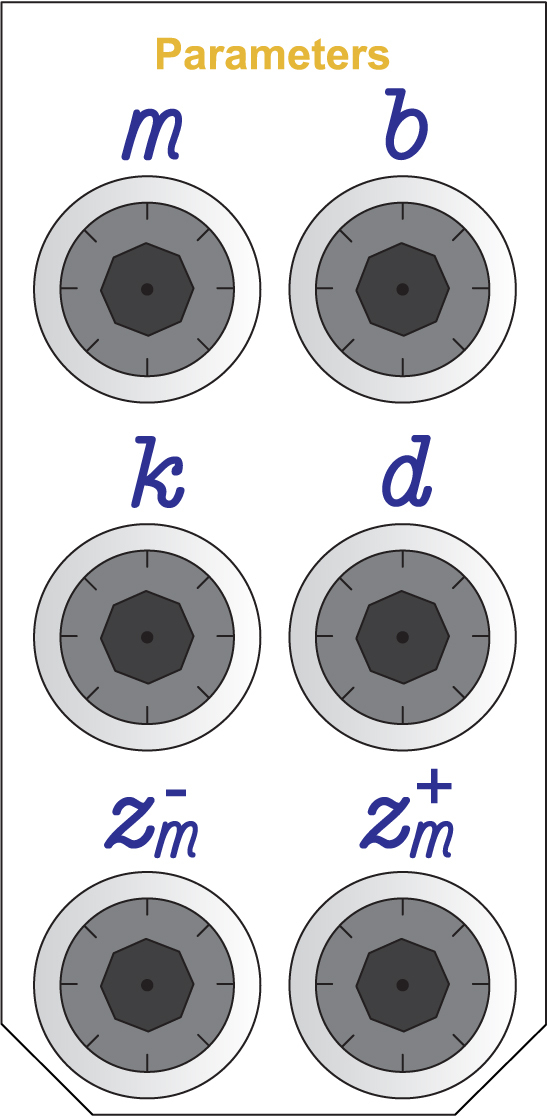
Knobs used for adjusting the equivalent physical parameters of the emulated system.

Fig. 8 shows the set of knobs used to adjust the main system parameters, allowing direct modification of the emulated mass, stiffness, damping, and nonlinearity characteristics.

### 2.6 Representative hardware implementation

The figures presented in this section correspond to a representative implementation of the proposed EMSD emulator. The illustrated hardware configuration demonstrates the feasibility of the approach and provides a practical reference for the overall organization of the system. The system architecture allows for extensions or modifications of the implemented dynamics, enabling the exploration of alternative configurations while preserving the same functional behavior.

The described implementation is provided as an example and does not limit the scope of the proposed emulator. Alternative electronic realizations, layouts, and physical configurations can be employed without altering the fundamental operation of the system.

Detailed electrical schematics, PCB layouts, and component level information required for the reproduction of the hardware are provided in the associated design files.

## 3 Design files summary

The design files provided with this article correspond to a representative hardware implementation for the proposed Electronic Mass–Spring–Damper (EMSD) emulator. These files are made publicly available to enable the reproduction, validation, and further development of the system for educational and research purposes.

The disclosed files describe one possible implementation of the emulator and are not intended to limit the scope of the underlying invention, which may be implemented using alternative electronic architectures, component selections, scaling factors, or physical arrangements while preserving the same functional behavior.

Electronic design files, additional 3D design for hardware assembly, user manual, simulation software, and data acquisition software are available in the public repository [24]. Table 1 summarizes the provided design files and their corresponding repository locations.

**Table 1:** Design files summary for a representative EMSD emulator implementation.

Design filename	File type	Open source license	Location of the file
Schematic_DT.pdf	Circuit schematic (PDF)	CERN- OHL-P	https://doi.org/10.17605/OSF.IO/GD3Z8
Schematic_M.pdf	Circuit schematic (PDF)	CERN- OHL-P	https://doi.org/10.17605/OSF.IO/GD3Z8
Schematic_DT.dch	Schematic (DipTrace Editable)	CERN-OHL-P	https://doi.org/10.17605/OSF.IO/GD3Z8
PCB_Layout_DT.dip	PCB layout (DipTrace Editable)	CERN-OHL-P	https://doi.org/10.17605/OSF.IO/GD3Z8
PCB_Gerber.zip	PCB Gerber files	CERN-OHL-P	https://doi.org/10.17605/OSF.IO/GD3Z8
Circuit_Simulator.ms13	Simulation file (Multisim)	CERN-OHL-P	https://doi.org/10.17605/OSF.IO/GD3Z8
Cover_Enclosure.pdf	Cover with knobs and terminals	CC-BY-4.0	https://doi.org/10.17605/OSF.IO/GD3Z8
Cover_Enclosure.cdr	Cover with knobs/terminals (Corel Draw Editable)	CC-BY-4.0	https://doi.org/10.17605/OSF.IO/GD3Z8
BOM.xlsx	Bill of materials	CERN-OHL-P	https://doi.org/10.17605/OSF.IO/GD3Z8
Quick_User_Guide.pdf	User manual	CC-BY-4.0	https://doi.org/10.17605/OSF.IO/GD3Z8
Base_Connectort_AC.stl	STL file for enclosure (3D printable)	CERN-OHL-P	https://doi.org/10.17605/OSF.IO/GD3Z8
Base_Enclosure.stl	STL file for enclosure (3D printable)	CERN-OHL-P	https://doi.org/10.17605/OSF.IO/GD3Z8
Cable_Comb_Ang.stl	STL file for enclosure (3D printable)	CERN-OHL-P	https://doi.org/10.17605/OSF.IO/GD3Z8
Cable_Comb_Large.stl	STL file for enclosure (3D printable)	CERN-OHL-P	https://doi.org/10.17605/OSF.IO/GD3Z8
Cable_Comb_Line.stl	STL file for enclosure (3D printable)	CERN-OHL-P	https://doi.org/10.17605/OSF.IO/GD3Z8
Spacers.stl	STL file for enclosure (3D printable)	CERN-OHL-P	https://doi.org/10.17605/OSF.IO/GD3Z8
EMSD_Data_Acquisition.vi	EMSD data acquisition file (LabVIEW Editable)	MIT License	https://doi.org/10.17605/OSF.IO/GD3Z8
EMSD_Simulator.slx	Simulation File (Simulink)	MIT License	https://doi.org/10.17605/OSF.IO/GD3Z8

### 3.1 Design files description:

• **Schematic_DT.pdf**: Schematic circuit corresponding to the representative implementation of EMSD, generated in DipTrace, provided for quick reference without requiring DipTrace to be installed. It can be opened with any PDF viewer.
• **Schematic_M.pdf**: Schematic circuit corresponding to the representative EMSD implementation, generated in Multisim, where the different modules of the circuit and their function are identified. It also serves as a quick reference without the need for Multisim to be installed. It can be opened with any PDF viewer.
• **Schematic_DT.dch**: Schematic circuit corresponding to the representative implementation of EMSD, created in DipTrace. It can be edited to incorporate additional modules. DipTrace version 4.2.0.1 or later is required.
• **PCB_Layout_DT.dip**: PCB layout corresponding to the representative schematic implementation described in “Schematic_DT.dch” schematic. It can be used to generate Gerber files for manufacturing and can also be helpful for circuit repair or maintenance. DipTrace Version 4.2.0.1 or later is required.
• **PCB_Gerber.zip**: Contains the industry-standard files required by a manufacturer to fabricate the PCB. They can be viewed with any free online program, such as the “KiCad Gerber Viewer (Online)” for example.
• **Circuit_Simulator.ms13**: Circuit simulator file used to test the design prior to fabrication; parameters are adjusted via virtual potentiometers, and signals are visualized using virtual oscilloscopes. Multisim 13.0 or later is required.
• **Cover_Enclosure.pdf**: Hardware cover layout showing the information available to the user and required to operate the hardware. Can be used as a guide for placing terminals and knobs. It can be opened with any PDF viewer.
• **Cover_Enclosure.cdr**: Editable CorelDRAW design for “Cover_Enclosure.pdf”. CorelDRAW 2021 or later is required.
• **BOM.xlsx**: Bill of Materials corresponding to the representative EMSD implementation, required to reproduce the hardware described in this work. Specifies the quantities, costs, and suggested suppliers. It can be opened with any compatible spreadsheet viewer.
• **Quick_User_Guide.pdf**: Contains the information necessary for a user without prior knowledge of the hardware to operate it. It can be opened with any PDF viewer.
• **3D Printed mechanical accessories (.stl files)**: This set of files includes auxiliary mechanical parts used in the representative EMSD implementation to support the PCB, secure the power supply, and organize internal wiring within the enclosure. These components facilitate cable and assembly management, but are not required for the functional operation of the emulator, and may be modified, replaced, or omitted according to the user’s enclosure or assembly preferences. The files can be opened and modified using standard 3D printing or CAD software.
• **EMSD_Data_Acquisition.vi**: LabVIEW program used for data acquisition through analog read and write tasks. From the front panel, the user can apply an input signal and visualize the hardware’s output signals in graphs. Once the VI is stopped, the acquired data are saved to a text file for further processing. LabVIEW 2014 or later is required.
• **EMSD_Simulator.slx**: Simulation of the mathematical model of the EMSD system used to compare its response with that of the representative hardware implementation, to validate its performance.

## 4 Bill of materials summary

The list of materials required for the reproduction of the hardware can be found in Table 2.

The list above includes the cost of higher quality components to ensure accurate performance and long durability of the hardware. However, it should be noted that there are more economical options available for some components, such as knobs or precision potentiometers; hence, equivalent components from other suppliers may be used.

**Table 2:** Bill of materials.

Component	Units	Cost per unit (USD)	Total cost (USD)	Source of materials	Material type
Enclosure 20X10X15 cm	1	23.75	23.75	Amazon.com	Plastic
Knobs	6	11.53	69.18	Newark.com	Metal-Plastic
10K Precision Potentiometer	6	11	66	Newark.com	Metal-Plastic
Cover	1	2	2		Metal-Plastic
Black block terminals	6	2.57	15.42	Digikey.com	Metal-Plastic
Red block terminals	6	2.57	15.42	Digikey.com	Metal-Plastic
AC Connector	1	1.06	1.06	Digikey.com	Metal-Plastic
AC Cable	1	2.59	2.59	Digikey.com	Copper-Plastic
Cable roll package	1	15.95	15.95	Digikey.com	Copper-Plastic
Dual symmetrical DC supply ±12 V/5V	1	17.7	17.7	Digikey.com	Electronic
PLA filament roll	1	17.77	17.77	Ebay.com	PLA
TL084CN Operational amplifier	4	0.85	3.4	Digikey.com	Semi-conductor
Resistor assortment	1	10.54	10.54	Digikey.com	Ceramic
Capacitor (1μF)	4	3.08	12.32	Digikey.com	Tantalum
1N4001 Diode	4	0.1	0.4	Digikey.com	Ceramic
PCB fabrication	1	5	5	https://www.allpcb.com/	Fiberglass

		Total	278.5		

## 5 Build instructions

This section describes the construction process of a representative hardware implementation of the proposed EMSD emulator. The instructions are intended to enable reproducibility while allowing flexibility in component selection, physical layout, and enclosure design. Detailed electrical schematics, PCB layouts, mechanical files, and component lists required for fabrication and assembly are provided in the associated design files and public repository [24].

**Fig. 9:**
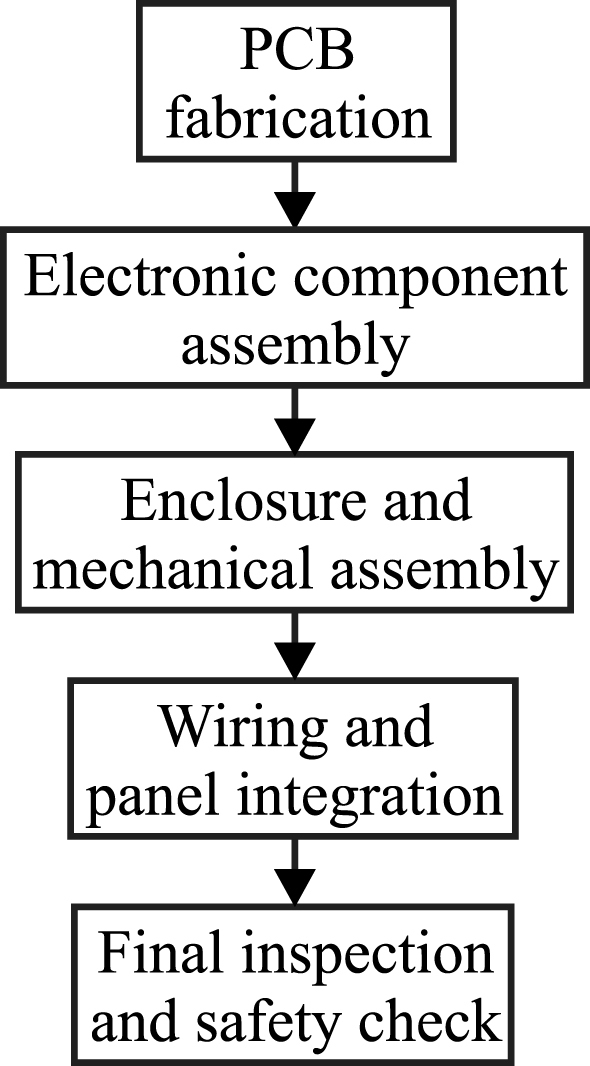
Representative assembly workflow for the EMSD emulator, illustrating the main construction stages from PCB fabrication to final inspection.

The assembly workflow corresponding to a representative implementation of the EMSD emulator is shown in Fig. 9. The diagram summarizes the main construction steps and provides a reference sequence for reproducing the hardware described in this work.

The following subsections describe a representative construction workflow consistent with the stages illustrated in Fig. 9.

### 5.1 PCB fabrication

The EMSD emulator is implemented on a two-layer printed circuit board. The PCB layout and manufacturing files (Gerber format) are provided in the design file summary and can be fabricated by any standard PCB manufacturer. No special manufacturing processes are required.

The PCB design corresponds to a representative realization of the proposed emulator architecture. Alternative PCB layouts or forms may be employed without altering the fundamental operation of the system, provided that the same functional blocks and signal interconnections are preserved.

### 5.2 Electronic components assembly

**Fig. 10:**
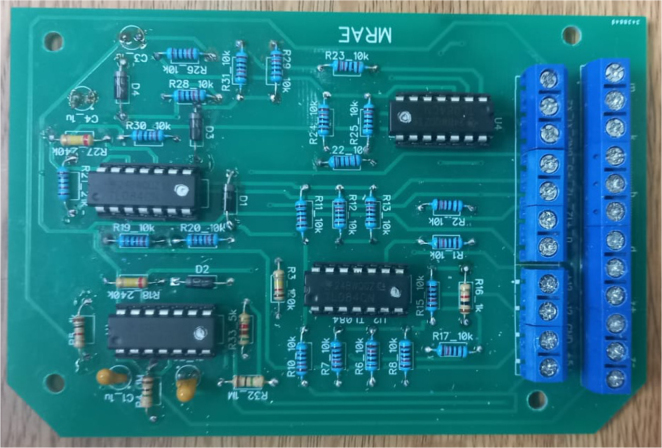
Assembled EMSD emulator printed circuit board corresponding to the reference hardware implementation.

Once the PCB has been fabricated, the electronic components listed in the bill of materials (BOM) should be soldered to the board (see Fig. 10). The BOM specifies the values, quantities, and suggested part types of the components to ensure the correct operation of the emulator.

The circuit is entirely based on continuous-time analog processing blocks and does not include any digital components or programmable devices. Adjustable parameters are implemented using precision potentiometers connected to the front panel, allowing for direct and intuitive tuning of the emulated physical parameters.

The design conditions in this representative implementation favor simplicity, transparency, and real-time operation. An analog architecture was selected to avoid discretization effects and numerical delays associated with digital implementations. Alternative realizations using different operational amplifiers, component tolerances, or modular extensions are possible, as long as the same functional behavior is maintained.

### 5.3 Enclosure integration and mechanical assembly

**Fig. 11:**
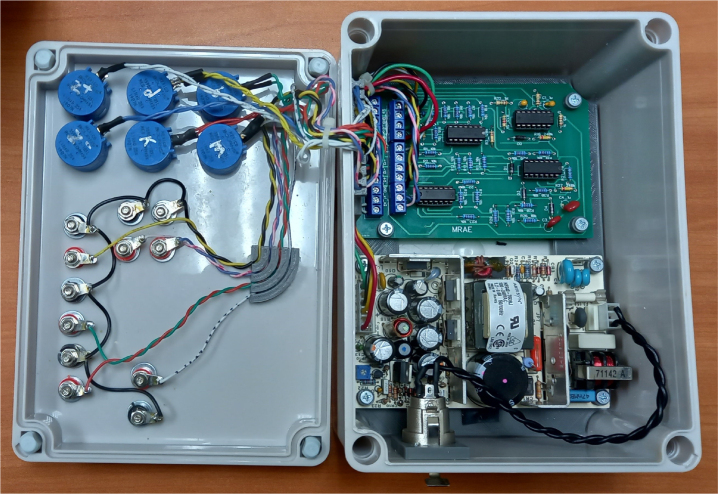
Internal view of a representative EMSD emulator assembly showing the relative placement of the PCB, power supply, and internal wiring.

After assembling the PCB, the hardware is integrated into a non-conductive enclosure suitable for laboratory use, (see Fig. 11). The enclosure accommodates the PCB, power supply, front panel controls, and signal terminals.

**Fig. 12:**
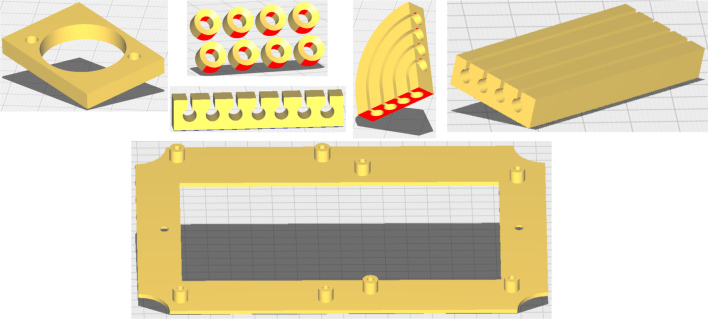
Representative 3D printed accessories used for mechanical support and internal cable organization. Design files are available in the repository.

Several auxiliary mechanical components were designed and manufactured using 3D printing to facilitate assembly and improve internal organization (see Fig. 12). These components include internal bases, spacers, and cable management elements used to secure the PCB, power supply, and wiring. Although not part of the electronic hardware itself, these elements improve robustness, repeatability, and ease of assembly. The corresponding STL files are provided in the repository and may be modified or replaced according to user requirements.

The front panel layout, including knob positions, terminal labeling, and parameter information, is provided as a printable and editable design file. This layout serves as a reference for assembling the user interface but does not impose a fixed physical configuration.

### 5.4 Wiring and final inspection

After mechanical assembly, the internal wiring should be completed according to the schematics provided and the enclosure layout. Make sure the polarity of the power supply connections is correct and that the signal and ground reference lines are properly routed.

Before operation, the assembled system should be visually inspected to verify the solder joints, connector integrity, and secure mounting of all components. A preliminary power-up test without external signal connections is recommended to confirm the correct supply voltages.

After wiring, the final assembly should match the front panel configuration shown in Fig. 1, where all user accessible controls and signal terminals are visible.

### 5.5 Safety considerations during assembly

The EMSD emulator operates from an internal power supply derived from an AC electrical network. The assembly and wiring should be performed with the device disconnected from the power source. Standard electrical safety practices should be followed when assembling the AC power input, including adequate insulation and mechanical cable support.

The enclosure is made of non-conductive material to provide electrical isolation. The internal ground reference used by the emulator should not be connected to the physical earth ground, as this may introduce interference and affect the system operation.

### 5.6 Visual and design references

Detailed schematics, PCB layouts, enclosure drawings, and mechanical files supporting the construction process are provided in the associated design files and repository. These visual resources complement the written instructions and allow for an accurate reproduction of the representative hardware implementation.

To facilitate mechanical integration and internal organization, a set of auxiliary components was designed and manufactured using 3D printing. These elements provide structural support and cable management during assembly and are not part of the core electronic hardware. Representative examples are shown in Fig. 12

## 6 Operation instructions

This section describes the necessary procedure for safely operating the EMSD emulator and performing reproducible experiments in an educational and research context. The description of the internal architecture and hardware design is presented in Section 2.

### 6.1 Power supply and safety considerations

As mentioned in Section 2, the EMSD emulator has an internal power supply designed to operate from a 120 VAC, 60 Hz power outlet. For operation, the user should connect the emulator to the electrical network through the power connector located on the side of the emulator.

The black terminals in the front panel (see Fig. 1) correspond to the system’s internal reference node and should not be connected to physical ground to avoid interference with signals. This reference should only be used as the common point for the EMSD’s input and output signals.

This device is housed in a non-conductive plastic enclosure, ensuring proper isolation of its low voltage internal circuits, thus eliminating the need for physical grounding.

Before powering on the device, it is recommended to verify that all external connections are correctly made. It is also recommended that the parameter adjustment knobs be set to their minimum position to avoid initial offset values in the amplifiers, which could cause amplifier saturation and significant transient effects in the response. This also ensures reproducibility in experiments with standard initial conditions.

### 6.2 Connection of input and output signals

The input signal u (see Fig. 1), can be generated using a function generator, an analog controller, or a data acquisition system.

The signals corresponding to position $x_{1}$ and velocity $x_{2}$ (see Fig. 1) can be read in real-time using an oscilloscope or a data acquisition system. They can also be used as feedback signals in closed loop control applications.

The black terminals are connected to the same node and should be used as a reference for any instruments that are externally connected to the EMSD emulator.

### 6.3 Additional voltage outputs

**Fig. 13:**
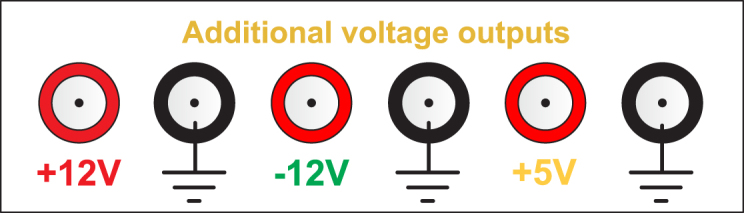
Additional voltage outputs.

The EMSD emulator front panel includes auxiliary regulated voltage terminals that provide +12V, −12V, and +5V outputs (see Fig. 13). These terminals allow users to power external analog and digital modules, such as operational amplifier circuits, signal conditioning stages, microcontrollers, etc. This feature facilitates rapid integration with external controllers and HIL applications.

#### 6.3.1 Example of connection with a data acquisition (DAQ) system

**Fig. 14:**
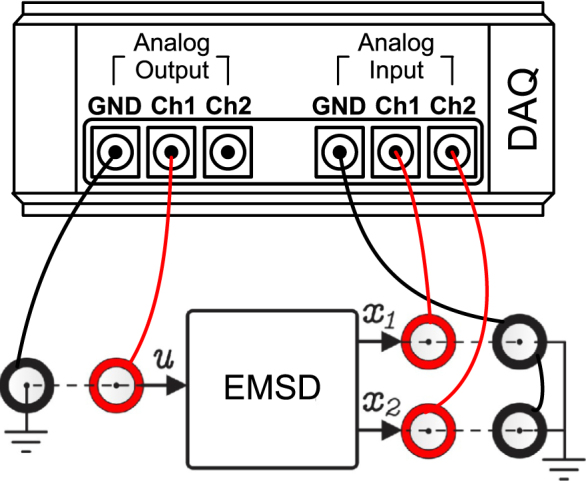
Example of emulator connection through a DAQ.

Fig. 14 shows an example of a connection using a data acquisition (DAQ) board. An analog output of the DAQ is used to generate the excitation signal of the emulator u, and the output signals of the emulator $x_{1}$ and $x_{2}$ are read through analog input channels.

#### 6.3.2 Example of connection with measurement instruments

**Fig. 15:**
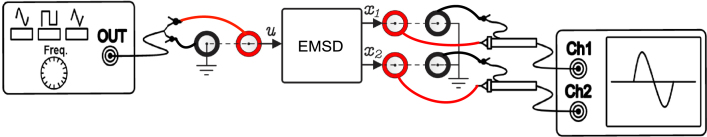
Example of emulator connection through measurement instruments.

Fig. 15 shows another example of how to connect the emulator using a function generator to produce the excitation signal u, and an oscilloscope to read the output signals $x_{1}$ and $x_{2}$.

### 6.4 System parameter adjustment

The emulated mechanical system parameters: mass(m), damping coefficient (b), spring stiffness (k), dry friction coefficient (d), and dead-zone thresholds ($Z_{m}^{+}$) and ($Z_{m}^{-}$); are adjusted using the six knobs located on the front panel (see Fig. 8).

Each knob tunes a 10 turn precision potentiometer. The relationship between the number of turns of each knob and the corresponding parameter value is presented in Table 3.

**Table 3:** Available value ranges for each parameter.

Number of	Mass value	Damping	Spring stiffness	Dry friction	Positive dead-	Negative dead-
knob turns	m (kg)	constant b (kg/s)	constant k (kg/s${}^{2}$)	coefficient d (N)	zone threshold $Z_{m}^{+}$ (N)	zone threshold $Z_{m}^{-}$ (N)
0	0.1	0	0	0	0	0
1	0.2	0.2	1	0.1	0.05	−0.05
2	0.3	0.4	2	0.2	0.10	−0.10
3	0.4	0.6	3	0.3	0.15	−0.15
4	0.5	0.8	4	0.4	0.20	−0.20
5	0.6	1.0	5	0.5	0.25	−0.25
6	0.7	1.2	6	0.6	0.30	−0.30
7	0.8	1.4	7	0.7	0.35	−0.35
8	0.9	1.6	8	0.8	0.40	−0.40
9	1.0	1.8	9	0.9	0.45	−0.45
10	1.1	2.0	10	1.0	0.50	−0.50

### 6.5 Conducting experiments

To ensure the reproducibility of the experiments, the following procedure is recommended.

1. Set all knobs to zero turns.
2. Power on the EMSD emulator (connect the power cable from the electrical network).
3. Gradually adjust the parameters to obtain the desired configuration.
4. If the nonlinear effects of the dead-zone and dry friction are to be included, adjust the parameters $Z_{m}^{+}$, $Z_{m}^{-}$, and d, respectively. Otherwise, these knobs can be set to a value of zero.

Once powered and configured, an input signal can be applied to excite the system’s dynamics. Common test signals include step and sinusoidal signals.

Position and velocity responses can be measured directly from terminals $x_{1}$ and $x_{2}$, respectively. Due to its fully analog implementation, the EMSD emulator operates in continuous-time, without any discretizations or software delays, making it particularly suitable for real-time experiments and HIL implementations.

### 6.6 System shutdown procedure

The recommended procedure for shutting down the system at the end of an experiment is as follows:

1. Set the input signal (u) to zero.
2. Power off the EMSD emulator (disconnect the power cable from the electrical network).
3. Safely disconnect any cables and external measurement or control equipment.

### 6.7 Care and maintenance

The emulator does not require special maintenance beyond what is typically required for electronic equipment. This includes proper handling, avoiding impacts, drops, and excessive exposure to humidity, dust, and temperature. It should be used according to its design specifications and stored in a clean, dry environment when not in use. By following the recommendations outlined in this section, stable performance, safe operation, and a long service life of the emulator are ensured.

Moreover, the emulator is maintenance-free. In case corrective maintenance is needed, refer to Sections 4 and 5 for internal components and their connections to identify potential causes of malfunction.

## 7 Validation and characterization

This validation aims to demonstrate both the accuracy and practical applicability of the proposed emulator under representative operating conditions. The purpose of this section is to validate the correct operation of the hardware and to characterize its performance when emulating linear dynamics, as well as certain nonlinear effects commonly found in mechanical systems, such as dry friction and dead-zone nonlinearities.

The performance of the EMSD emulator was validated by comparing its experimental response with the numerical simulation of the same mathematical model emulated under identical parametric conditions.

### 7.1 Experimental setup

Experimental data were obtained using a National Instruments USB6001 data acquisition device (DAQ). The USB6001 is a low-cost, USB-based DAQ that provides 12 bit analog input and output channels. For the experimental data presented in this work, the DAQ was configured to generate and acquire analog signals within the ±10 V range, with a fixed sampling period of 0.01 s.

The reference numerical simulation was implemented in Simulink using a first order ODE solver (Euler) with a fixed step size of 0.01 s. The use of a fixed step solver that matches the DAQ sampling period ensures temporal alignment between the simulated and experimental signals and avoids the need for interpolation effects during comparison.

### 7.2 Error metrics

To quantitatively evaluate the discrepancy between the simulated and experimental responses, four complementary error metrics were employed. A global metric based on the energy of the error signal was used to evaluate the overall deviation between both responses. In addition, two point-wise metrics (the Root Mean Square Error (RMSE) and the maximum absolute error ($e_{\max}$)) were considered to capture the average and worst-case differences, respectively. These metrics are defined based on discretely sampled signals obtained from both numerical simulations and experimental measurements, ensuring a consistent comparison between the two responses.

Let $x_{s}\left(k\right)$ denote the simulated response and $x_{e}\left(k\right)$ correspond to the experimental response. Where k denotes the discrete-time sample index, since both the simulated and experimental signals are obtained from sampled data via numerical simulation and data acquisition.

The energy-based error metric is defined as:

(3) $$\text{ErrorEnergy}=\overset{n}{\underset{k=1}{\sum }}{\left[x_{s}\left(k\right)-x_{e}\left(k\right)\right]}^{2}.$$

where n represents the number of samples. Similarly, the energy of the simulation signal is computed as

(4) $$\text{SimEnergy}=\overset{n}{\underset{k=1}{\sum }}{\left[x_{s}\left(k\right)\right]}^{2}.$$

Using the previous definitions (Eq. (3)) and (Eq. (4)), the normalized error energy is calculated as

(5) $$\text{\%Error}=\frac{\text{ErrorEnergy}}{\text{SimEnergy}}\left(100\right).$$

This metric physically indicates the proportion of the overall system response that is affected by discrepancies between the emulator and the numerical model. Low values imply that the error contributes minimally to the total signal energy.

The point-wise error metrics are defined as:

(6) $$e_{\max}=\underset{k}{\max}\left|x_{s}\left(k\right)-x_{e}\left(k\right)\right|$$

(7) $$RMSE=\sqrt{\frac{1}{n}\overset{n}{\underset{k=1}{\sum }}{\left(x_{s}\left(k\right)-x_{e}\left(k\right)\right)}^{2}}$$

To express the RMSE values in relative terms, the error was normalized using the motion range of each signal, computed as the difference between its maximum and minimum observed values. The relative RMSE was therefore defined as:

(8) $$\text{Relative\_RMSE}=\frac{\text{RMSE}}{x_{\max}-x_{\min}}$$

This normalization provides a scale independent measure of the average discrepancy between the simulated and experimental responses. In particular, the Relative RMSE represents the average error expressed as a percentage of the signal range, allowing a more intuitive interpretation of the deviation with respect to the amplitude of the system motion.

### 7.3 Validation experiments

To validate the performance of the emulator, several scenarios were considered, as described below. Although both position and velocity signals are available in the emulator, the analysis in the following cases focuses primarily on the position response, as it represents the final output of the system and captures the overall dynamic behavior. Even though both integration stages are configured similarly, the velocity signal coming out of the first integrator is more sensitive to noise and small discrepancies in the electronic components, as it contains higher-frequency components associated with its role as an intermediate state. In contrast, the position signal, obtained through an additional integration stage, exhibits a smoothing effect that attenuates high-frequency noise, resulting in a more stable and reliable signal for system validation purposes. Therefore, the position signal provides a more robust basis for quantitative comparison between the emulator and the numerical model. For completeness, both signals are presented in Case 1, while the remaining cases focus on position to highlight the effect of nonlinearities on the overall system response.

#### 7.3.1 Case 1: Linear dynamics without nonlinear effects

**Fig. 16:**
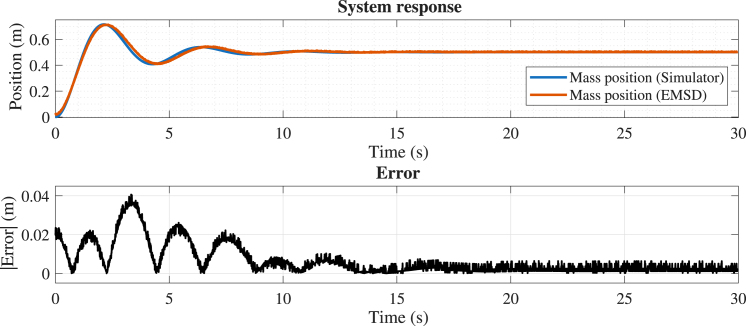
Comparison between the simulated ($x_{1s}$) and experimental ($x_{1e}$) mass position.

**Fig. 17:**
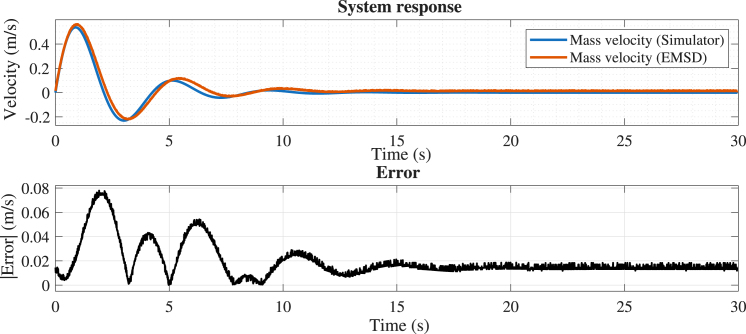
Comparison between the simulated ($x_{2s}$) and experimental ($x_{2e}$) mass velocity.

In the first experiment, dry friction and dead-zone effects were disabled. The mass, spring, and damping parameters were set at 0.8, 2, and 0.6, respectively. The position error (see Fig. 16) remains very low, corresponding to less than 2% of the motion range, with maximum deviations occurring primarily during the initial transient. These results indicate excellent agreement between the analog implementation and the numerical model under linear operating conditions. For the velocity signal (see Fig. 17), slightly larger deviations are observed; nevertheless, the error remains bounded within a small fraction of the signal range (below 5%), confirming a consistent and stable dynamic response.

#### 7.3.2 Case 2: Linear dynamics with dry friction nonlinearity

**Fig. 18:**
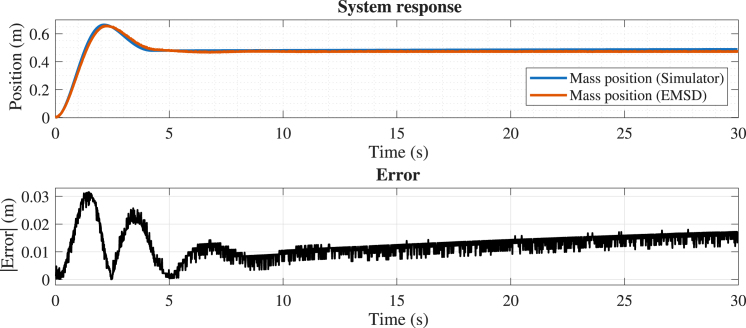
Comparison between the simulated ($x_{1s}$) and experimental ($x_{1e}$) mass position, considering dry friction.

The second experiment considers dry friction while keeping the dead-zone parameter equal to zero. The mass, spring, and damping parameters were set at 0.8, 2, and 0.6, respectively, and the dry friction coefficient was set at 0.7. The position error (see Fig. 18) remains low, below approximately 2% of the motion range, with discrepancies mainly concentrated during transient responses. These results confirm that the emulator maintains high accuracy even in the presence of the dry friction effect.

#### 7.3.3 Case 3: Linear dynamics with dead-zone nonlinearity

**Fig. 19:**
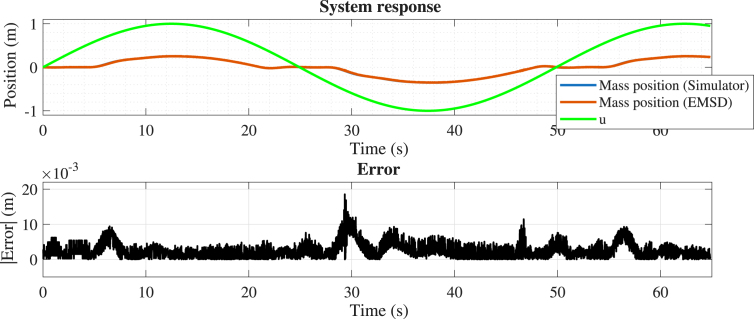
Comparison between the simulated ($x_{1s}$) and experimental ($x_{1e}$) mass position, considering dead-zone.

In this case, only the dead-zone nonlinearity was enabled. The mass, spring, and damping parameters were set at 0.8, 2, and 0.6, respectively, while the dead-zone thresholds were defined as 0.5 and −0.3. The position error (see Fig. 19) is minimal, remaining well below 1% of the motion range, with only small deviations observed near transitions across the threshold boundaries. This indicates excellent agreement and precise reproduction of the dead-zone behavior.

#### 7.3.4 Case 4: Combined effects of dry friction and dead-zone

**Fig. 20:**
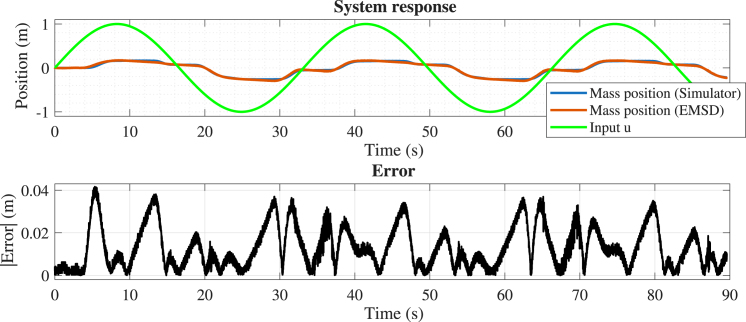
Comparison between the simulated ($x_{1s}$) and experimental ($x_{1e}$) mass position, considering dry friction and dead-zone.

The final experiment evaluates the emulator performance with both dry friction and dead-zone nonlinearities enabled simultaneously. The mass, spring, and damping parameters were set at 0.8, 2, and 0.6, respectively. The dry friction coefficient was set at 0.7, and the dead-zone thresholds were set at 0.5 and −0.3. The position error (see Fig. 20) increases compared to previous cases but remains below 4% of the motion range, with discrepancies mainly observed during transitions between nonlinear regimes. These results confirm that the emulator preserves both the qualitative and quantitative behavior of the system under combined nonlinear effects.

To provide a comprehensive comparison across all experimental scenarios, Table 4 summarizes the main quantitative error metrics obtained for each case.

The quantitative metrics presented in Table 4 confirm that the emulator closely reproduces the simulated dynamics across all test cases. In particular, the Relative RMSE for the position signal remains below 2% in most scenarios, indicating a high level of accuracy in both transient and steady-state responses.

**Table 4:** Summary of error metrics for all test cases.

Case	Signal	Error energy (%)	RMSE	Relative RMSE (%)	$e_{max}$
Case 1	Position	0.04	0.010217	1.43	0.040651
	Velocity	4.63	0.023804	4.43	0.077900
Case 2	Position	0.08	0.013388	2.01	0.031545
Case 3	Position	0.03	0.003127	0.52	0.018527
Case 4	Position	1.16	0.016634	3.90	0.041611

As expected, slightly larger deviations are observed in the velocity signal in Case 1, which is inherently more sensitive to noise and small discrepancies in the analog components. Nevertheless, these differences remain bounded and do not significantly affect overall system behavior.

Across all scenarios, the error levels remain consistently low, even in the presence of nonlinear effects such as dry friction and dead-zone characteristics. This demonstrates that the proposed emulator maintains stable performance and provides a reliable and robust physical representation of the modeled system under varying operating conditions.

### 7.4 Performance characterization and analysis

The results obtained from the four experimental scenarios demonstrate that the proposed emulator accurately reproduces the dynamic behavior of the system under both linear and nonlinear operating conditions. As summarized in Table 4, the Relative RMSE for the position signal remains below 4% in all cases, and below 2% in most scenarios, indicating a high level of agreement between the experimental and simulated responses in both transient and steady-state regimes.

From a global perspective, the normalized error energy remains consistently low across all test cases, indicating that only a small fraction of the total signal energy is associated with the error. This confirms that the discrepancies between the emulator and the numerical model have a negligible impact on the overall system dynamics.

In the linear case (Case 1), the lowest error levels are observed, as expected, since the system dynamics are fully described by linear components. When nonlinear effects are introduced, a slight increase in error is observed. In particular, the presence of dry friction (Case 2) introduces small deviations during transient responses, while the dead-zone nonlinearity (Case 3) produces localized discrepancies near threshold transitions. The combined case (Case 4) exhibits the highest error levels, although the Relative RMSE remains below 4%, confirming that the emulator preserves both qualitative and quantitative behavior even under more complex nonlinear conditions.

The complementary error metrics provide additional insight into the emulator performance. While the Relative RMSE captures the average deviation relative to the signal amplitude, the maximum absolute error reflects worst-case discrepancies, which remain bounded in all cases. The normalized error energy, on the other hand, provides a global measure of deviation, confirming that the error contribution is small compared to the overall signal energy.

The observed discrepancies can be attributed to several practical factors inherent to analog implementations, including component tolerances, offset voltages, and noise. These factors primarily affect transient responses and intermediate signals, such as the velocity, which is more sensitive to high-frequency components. In contrast, the position signal benefits from an additional integration stage, resulting in a smoothing effect that reduces the impact of high-frequency noise and leads to a more stable and reliable observable for validation.

Overall, the consistency of the error metrics across all scenarios demonstrates that the proposed emulator provides a robust, accurate, and physically meaningful implementation of the modeled system, making it suitable for both educational and experimental applications involving linear and nonlinear dynamic systems.

### 7.5 Capabilities and limitations

Capabilities:

• Emulation of linear mass–spring–damper systems with adjustable parameters.
• Representation of dry friction and dead-zone nonlinearities.
• Quantitative performance validation through error metrics.
• Real-time parameter adjustment via front panel controls.
• Access to the complete state vector for measurement and controller design.
• Compatibility with basic laboratory equipment.
• Useful for hardware-in-the-loop (HIL) testing.

Limitations:

• Accuracy is limited by electronic component tolerances and electronic noise.
• High frequency dynamics are limited by the bandwidth of the analog circuits.
• The effects of nonlinear terms are approximated and therefore may exhibit deviations under certain operating conditions.

### 7.6 Conclusions

This work presents an open-source electronic emulator of a mass–spring–damper mechanical system with configurable nonlinear dynamics, implemented using continuous-time analog processing blocks. The proposed hardware enables real-time experimentation with linear and nonlinear dynamic behaviors, including dry friction and dead-zone effects, commonly encountered in real systems.

The experimental results demonstrate that the emulator accurately reproduces the system dynamics across a range of operating conditions. Quantitative validation shows low error levels, with Relative RMSE values below 4% in all test cases and below 2% in most scenarios. This result highlights the robustness of the proposed approach, even in the presence of nonlinear effects.

The emulator is designed to be reproducible, modular, and accessible, making it suitable for educational laboratories, control research, and hardware-in-the-loop (HIL) applications. By combining transparent analog implementation, user-adjustable parameters, and publicly available design files, the proposed platform provides a practical and flexible tool for hands-on exploration of system dynamics and control concepts.

Overall, the proposed system offers a low-cost and physically interpretable alternative to traditional experimental platforms, bridging the gap between theoretical modeling and real world implementation while facilitating further extensions and adaptations by the research and educational communities.

## CRediT authorship contribution statement

**Ernesto V. Gonzalez-Solis:** Writing – original draft, Visualization, Validation, Methodology, Investigation, Conceptualization. **David I. Rosas-Almeida:** Writing – review & editing, Validation, Methodology, Investigation, Conceptualization.

## Funding

This research did not receive specific grants from funding agencies in the public, commercial, or non-profit sectors.

## Declaration of competing interest

The authors declare that they have no known competing financial interests or personal relationships that could have appeared to influence the work reported in this paper.

## Appendix A Supplementary data

MMC S1 Supplementary material includes design files, schematics, PCB layouts, and 3D models associated with the open-source hardware implementation of the proposed system. MMC S1
