# Supplementary material for: Open-source Electronic Mass–Spring–Damper emulator with nonlinear dynamics for education and control research
Source: HardwareX. 2026 May 8;26:e00785. doi: 10.1016/j.ohx.2026.e00785 (PMC13187592; doi:10.1016/j.ohx.2026.e00785)
Supplement: MMC S1 — Supplementary material includes design files, schematics, PCB layouts, and 3D models associated with the open-source hardware implementation of the proposed system. [file mmc1.zip › 1.zip!/Schematic_DT.pdf]

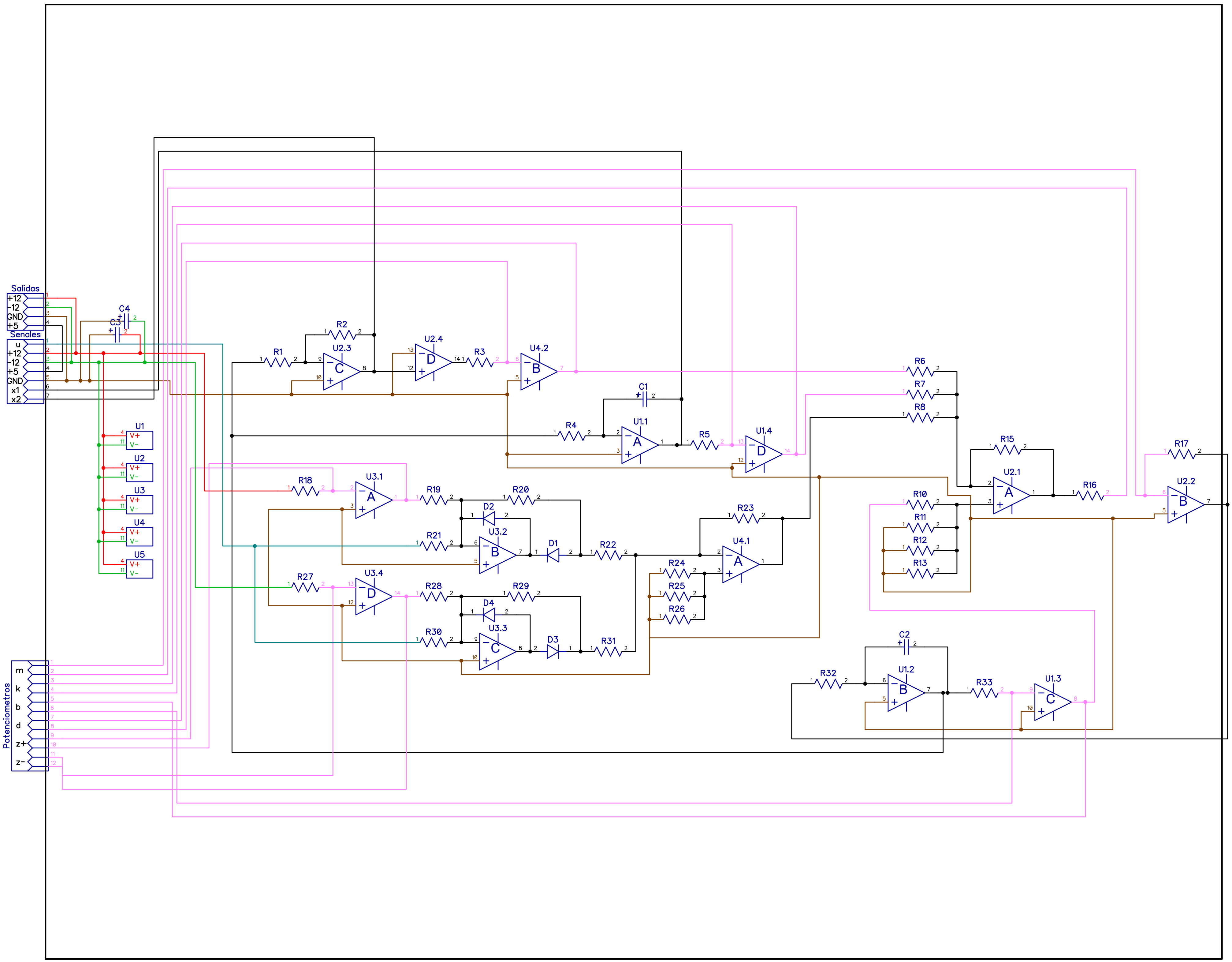
Salidas
+12
-12
GND
+5
Senales
u
+12
-12
+5
GND
x1
x2
C3
C4
U1
U2
U3
U4
U5
V+
V-
Potenciometros
m
k
b
d
z+
z-
R1
R2
U2.3
C
U2.4
D
R3
U4.2
B
C1
R4
U1.1
A
R5
U1.4
D
R6
R7
R8
R15
U2.1
A
R16
R17
U2.2
B
R10
R11
R12
R13
R18
U3.1
A
R19
R20
D2
R21
U3.2
B
D1
R22
R23
U4.1
A
R24
R25
R26
R27
U3.4
D
R28
R29
D4
R30
U3.3
C
D3
R31
C2
R32
U1.2
B
R33
U1.3
C
