# Supplementary material for: Open-source Electronic Mass–Spring–Damper emulator with nonlinear dynamics for education and control research
Source: HardwareX. 2026 May 8;26:e00785. doi: 10.1016/j.ohx.2026.e00785 (PMC13187592; doi:10.1016/j.ohx.2026.e00785)
Supplement: MMC S1 — Supplementary material includes design files, schematics, PCB layouts, and 3D models associated with the open-source hardware implementation of the proposed system. [file mmc1.zip › 1.zip!/Schematic_M.pdf]

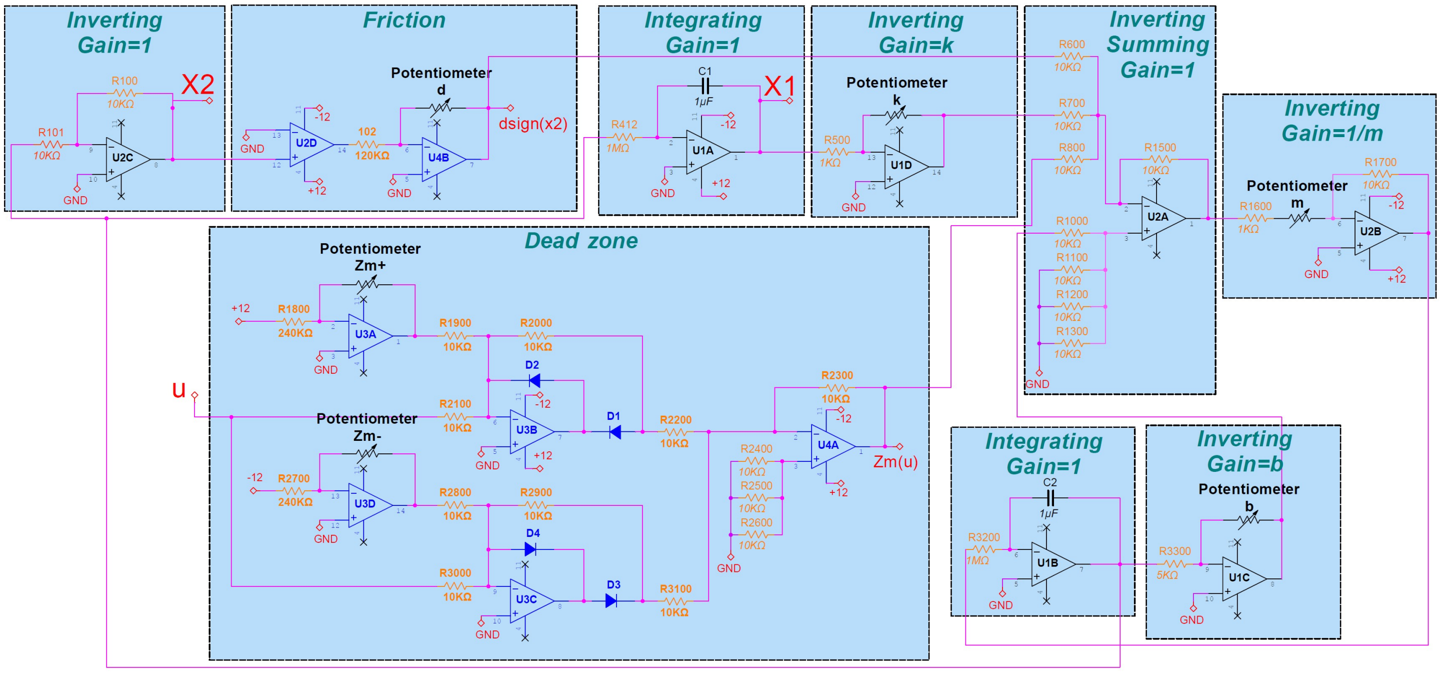
Inverting Gain=1
Friction
Integrating Gain=1
Inverting Gain=k
Inverting Summing Gain=1
Inverting Gain=1/m
Dead zone
Integrating Gain=1
Inverting Gain=b
X2
X1
dsign(x2)
u
Zm(u)
Potentiometer d
Potentiometer k
Potentiometer m
Potentiometer Zm+
Potentiometer Zm-
Potentiometer b
R100 10KΩ
R101 10KΩ
102 120KΩ
R412 1MΩ
C1 1μF
R500 1KΩ
R600 10KΩ
R700 10KΩ
R800 10KΩ
R1000 10KΩ
R1100 10KΩ
R1200 10KΩ
R1300 10KΩ
R1500 10KΩ
R1600 1KΩ
R1700 10KΩ
R1800 240KΩ
R1900 10KΩ
R2000 10KΩ
R2100 10KΩ
R2200 10KΩ
R2300 10KΩ
R2400 10KΩ
R2500 10KΩ
R2600 10KΩ
R2700 240KΩ
R2800 10KΩ
R2900 10KΩ
R3000 10KΩ
R3100 10KΩ
R3200 1MΩ
C2 1μF
R3300 5KΩ
U2C
U2D
U4B
U1A
U1D
U2A
U2B
U3A
U3B
U3D
U3C
U4A
U1B
U1C
D1
D2
D3
D4
GND
+12
-12
